# The Ferric Citrate Uptake System Encoded in a Novel *bla*_CTX–M–3_- and *bla*_TEM–1_-Harboring Conjugative Plasmid Contributes to the Virulence of *Escherichia coli*

**DOI:** 10.3389/fmicb.2021.667782

**Published:** 2021-05-26

**Authors:** Wen-Chun Huang, Min-Yi Wong, Ssu-Han Wang, Masayuki Hashimoto, Meng-He Lin, Mei-Feng Lee, Jiunn-Jong Wu, Ming-Cheng Wang, Wei-Hung Lin, Shuen-Lin Jeng, Jiun-Ling Wang, Ya-Lei Chen, Ching-Hao Teng

**Affiliations:** ^1^Institute of Molecular Medicine, College of Medicine, National Cheng Kung University, Tainan, Taiwan; ^2^Institute of Basic Medical Sciences, College of Medicine, National Cheng Kung University, Tainan, Taiwan; ^3^Center of Infectious Disease and Signaling Research, National Cheng Kung University, Tainan, Taiwan; ^4^Department of Biotechnology, National Kaohsiung Normal University, Kaohsiung, Taiwan; ^5^Department of Biotechnology and Laboratory Science in Medicine, School of Biomedical Science and Engineering, National Yang Ming Chiao Tung University, Taipei, Taiwan; ^6^Division of Nephrology, Department of Internal Medicine, National Cheng Kung University Hospital, College of Medicine, National Cheng Kung University, Tainan, Taiwan; ^7^Department of Internal Medicine, National Cheng Kung University Hospital, College of Medicine, National Cheng Kung University, Tainan, Taiwan; ^8^Department of Statistics, Institute of Data Science, Center for Innovative FinTech Business Models, National Cheng Kung University, Tainan, Taiwan; ^9^Department of Internal Medicine, National Cheng Kung University Hospital, Tainan, Taiwan; ^10^Department of Medicine, College of Medicine, National Cheng Kung University, Tainan, Taiwan; ^11^Center of Allergy and Clinical Immunology Research (ACIR), National Cheng Kung University, Tainan, Taiwan

**Keywords:** iron uptake system, *bla*_CTX–M–3_, ESBL, bacteremia, urinary tract infections, ferric citrate, *E. coli*, conjugative plasmid

## Abstract

*Escherichia coli* is one major cause of bacterial infections and can horizontally acquire antimicrobial resistance and virulence genes through conjugation. Because conjugative plasmids can rapidly spread among bacteria of different species, the plasmids carrying both antimicrobial resistance and virulence genes may pose a significant threat to public health. Therefore, the identification and characterization of these plasmids may facilitate a better understanding of *E. coli* pathogenesis and the development of new strategies against *E. coli* infections. Because iron uptake ability is a potential virulence trait of bacteria, we screened for *E. coli* conjugative plasmids able to confer both iron uptake ability and ampicillin resistance. The plasmid pEC41, which was derived from the bacteremia clinical isolate EC41, was identified. EC41, which carried the *fimH*27 allele, belonged to sequence type (ST) 405 and phylogroup D. According to the sequencing analyses, pEC41 was 86 kb in size, and its backbone structure was almost identical to that of another highly conjugative plasmid, pCTX-M3, in which the extended-spectrum β-lactamase gene *bla*_CTX–M–3_ was originally identified. pEC41 carried *bla*_CTX–M–3_ and *bla*_TEM–1_. The ferric citrate uptake (*fec*) system was identified in pEC41 and was responsible for conferring iron uptake ability. The *fec* system contributes to the pathogenesis of EC41 in systemic infections but not in urinary tract infections (UTIs). However, this system promoted competitive fitness of a cystitis-associated clinical isolate to colonize urinary tracts. Additionally, the distribution of the *fec* system was related to *E. coli* isolates associated with human bacteremia and UTIs. In summary, the present study identified a novel conjugative plasmid, pEC41, which conferred both antimicrobial resistance and an extra iron uptake ability to *E. coli*. The iron uptake ability was encoded in the *fec* system and contributed to *E. coli* pathogenesis. This study is the first to show that the *fec* system is a virulence factor in *E. coli*.

## Introduction

*Escherichia coli* is one of the major Gram-negative etiological causes of bacteremia and urinary tract infections (UTIs) (Medina and Castillo-Pino, 2019; Bonten et al., 2020; Wagenlehner et al., 2020). *E. coli* can obtained antimicrobial resistance and virulence factors through horizontal gene transfer (HGT) (Paauw et al., 2009; Wang et al., 2011; Johnson and Lang, 2012). Conjugative plasmids, which are one of the key agents of HGT, can spread efficiently and broadly among distinct bacterial species through conjugation (Ilangovan et al., 2015; Partridge et al., 2018). Therefore, the plasmids carrying both antimicrobial resistance and virulence genes may pose a significant threat to public health (Paauw et al., 2009; Wang et al., 2011; Johnson and Lang, 2012). The identification and characterization of these plasmids would provide potential strategies to tackle the worldwide public health issues. However, identifying the virulence factors carried by the plasmids could be a challenge because some of the pathogenic effects of these factors are only observed in specific tissues, and the potential presence of other factors with similar functions in the same host bacteria may hinder the discovery of such plasmid-carried virulence factors (Cusumano et al., 2010; Garcia et al., 2011; Huang et al., 2020).

Iron uptake ability is a potential virulence trait for invading bacteria to survive in hosts, where the bioavailability of iron is restricted (Hood and Skaar, 2012). Iron uptake systems are often identified in bacterial chromosomes, while plasmid-encoded systems are relatively rare (Ramirez et al., 2014). However, based on *in silico* analysis, various potential iron uptake genes have been detected in plasmid sequences, suggesting that many plasmid-encoded iron uptake systems that contribute to bacterial virulence have yet to be identified. Siderophore systems, including enterobactin, salmochelin, aerobactin, and yersiniabactin, have been utilized by pathogenic bacteria to retrieve ferric iron (Fe^3+^) as an iron source in hosts and thus contribute to the virulence of the bacteria. On the other hand, the *fecIRABCDE* (*fec*) system also enables bacteria to retrieve Fe^3+^ through the uptake of ferric citrate (Braun and Mahren, 2005). However, researchers have not clearly determined whether this system contributes to any infections. This system is composed of the upstream *fecIR* operon and the downstream *fecABCDE* operons. The *fecABCDE* operons encode the ability to take up ferric citrate, while *fecIR* is responsible for regulating the transcription of *fecABCDE*. The transcription of *fecABCDE* and *fecIR* is repressed by iron-bound Fur. The expression of *fecIR* is induced under iron-limiting conditions, while the expression of *fecABCDE* requires both the induction of iron limitation and the presence of ferric citrate (Braun and Mahren, 2005). Given the constant presence of citrate in body fluids and the high affinity of this compound for ferric iron (Kirejczyk et al., 2014; Costello and Franklin, 2016), the *fec* system may contribute to the virulence of bacteria during infections.

In the present study, we aimed to identify and characterize conjugative plasmid-encoded iron uptake systems that can contribute to the pathogenesis of *E. coli* infections. We started by performing an *in vitro* screen of clinical isolate-derived conjugative plasmids that increase the iron uptake ability of bacterial hosts. Because pathogenic *E. coli* usually harbors multiple iron systems to cope with iron-limited environments, the coexistence of functionally redundant iron uptake factors in the bacteria may interfere with the screening. To avoid such potential interference, ampicillin-resistant conjugative plasmids in clinical strains were conjugally transferred to an iron uptake-defective commensal *E. coli* K12 strain whose enterobactin-dependent uptake system was inactivated. The resulting transconjugants were then subjected to a screen for the upregulated abilities to acquire iron. A plasmid encoding the *fec* system was identified, and this system was found to contribute to the virulence of *E. coli*. In addition, this plasmid carried genes encoding an extended-spectrum β-lactamase (ESBL) and a broad-spectrum β-lactamase.

## Materials and Methods

### Bacterial Strains, Plasmids, Growth Conditions, and Reagents

The *E. coli* strains utilized for genetic manipulation are shown in Table 1. Donor clinical *E. coli* isolates were collected from National Cheng Kung University Hospital (NCKUH), Taiwan (NCKUH IRB approval no. A-ER-104-313). For the isolates used in the molecular epidemiology study, the fecal and UTI-associated *E. coli* isolates were described previously (Mao et al., 2012), and bacteremia-associated *E. coli* were collected from the blood specimens of patients at NCKUH between October and December of 2005. The bacteria were grown on Luria-Bertani (LB) (1% tryptone, 0.5% yeast extract, and 1% NaCl) broth or agar with appropriate antimicrobial s at 37°C unless otherwise indicated. The reagents and antimicrobial s were purchased from Sigma-Aldrich, St. Louis, MO, United States unless otherwise indicated.

**TABLE 1:** Bacterial strains and plasmids used in this study.

Strains or plasmids	Description	Known AR markers^a^	Sources
**Strains**			
EC41	An *E. coli* strain isolated from the blood of a patient	Ap, Str, Km	This study
A487	An *E. coli* strain isolated from the urine of a patient with cystitis		Mao et al., 2012
CFT073^str^	A spontaneous streptomycin-resistant mutant of the urosepsis-causing *E. coli* strain CFT073	Str	This study
UTI89^str^	A spontaneous streptomycin-resistant mutant of the cystitis-causing *E. coli* strain UTI89	Str	This study
MG1655^str^	A spontaneous streptomycin-resistant mutant of *E. coli* K12 MG1655	Str	This study
IP10	An *entA* mutant of MG1655 with an insertion of a kanamycin resistance cassette in the *entA* locus	Km	This study
MP10	An *entA* mutant of MG1655^str^	Str	This study
MP10LA	A *lacZ* mutant of MP10 with an insertion of a chloramphenicol resistance cassette in the *lacZ* locus	Str, Cm	This study
ELA41	MP10LA harboring pEC41	Str, Cm, Ap	This study
IP10/pEC41	IP10 harboring pEC41	Km, Ap	This study
MP10/pEC41	MP10 harboring pEC41	Str, Ap	This study
Δ*fec*-MP10	A *fec*^MG1655^ deletion mutant of MP10 with an insertion of a gentamicin resistance cassette in the chromosomal *fec*^MG1655^ locus	Str, Gm	This study
MP10/pEC41Δ*fec*	MP10 harboring pEC41Δ*fec*	Str, Ap, Cm	This study
Δ*fec*-MP10/pEC41	Δ*fec*-MP10 harboring pEC41	Str, Ap, Gm	This study
Δ*fec*-MP10/pEC41Δ*fec*	Δ*fec*-MP10 harboring pEC41Δ*fec*	Str, Ap, Gm, Cm	This study
Δ*fec*^pEC41^-EC41	A *fec*^EC41^ deletion mutant of EC41 with an insertion of chloramphenicol resistance cassette in the *fec*^EC41^ locus	Ap, Cm	This study
A487/pEC41	A487 harboring pEC41	Ap	This study
A487/pEC41Δ*fec*	A487 harboring pEC41Δ*fec*	Ap, Cm	This study
CFT073^str^/pEC41	A spontaneous streptomycin-resistant strain of *E. coli* CFT073 harboring pEC41	Str, Ap	This study
UTI89^str^/pEC41	A spontaneous streptomycin-resistant strain of *E. coli* UTI89 harboring pEC41	Str, Ap	This study
Plasmids			
pEC41	The plasmid isolated from EC41	Ap	This study
pEC41Δ*fec*	A *fec*^EC41^ deletion mutant of pEC41 with an insertion of chloramphenicol resistance cassette in the *fec*^EC41^ locus	Ap, Cm	This study

*^a^AR markers, antimicrobial resistance markers commonly used in laboratories; Ap, ampicillin; Str, streptomycin; Km, kanamycin; Cm, chloramphenicol; Gm, gentamicin.*

### Conjugation

To collect ampicillin-resistant conjugative plasmids from clinical *E. coli* isolates by using the chloramphenicol-resistant *E. coli* strain MP10LA (Table 1) as the recipient through conjugation, clinical isolates that exhibited ampicillin resistance and chloramphenicol susceptibility (i.e., isolates able to grow in medium containing 50 μg/ml of ampicillin, but unable to grow in medium containing 15 μg/ml of chloramphenicol) was selected as donors. Equal volumes of overnight cultures of the donor clinical isolates and the recipient strain MP10LA were mixed and spread on LB agar to capture conjugative plasmids conferring ampicillin resistance from clinical *E. coli* isolates. After 6 h of incubation, the bacteria were suspended in LB broth and plated on LB agar with chloramphenicol and ampicillin to select against the donor and recipient bacteria. Therefore, the resulting transconjugants, which were MP10LA strains that transconjugatively obtained ampicillin-resistant plasmids, survived and grew on LB agar.

The pEC41-harboring donor strains (EC41, ELA41, and IP10/pEC41) were mixed with recipients for the conjugative transfer of pEC41. Ampicillin and a proper antimicrobial were used to select against the recipient and donor strains, respectively and to identify the resulting transconjugants.

### *E. coli* Typing

The sequence type (ST), phylogroup, and *fimH* type of EC41 were determined using the PubMLST.org website, ClermonTyping, and FimTyper, respectively, as described previously (Roer et al., 2017; Beghain et al., 2018; Jolley et al., 2018).

### Mutant Construction

The *E. coli* mutants utilized in the present study were constructed using λ red mediated recombineering as described previously (Datsenko and Wanner, 2000; Hsu et al., 2020).

To construct the *fec* system deletion mutant of the plasmid pEC41, pEC41 was conjugatively transferred to MG1655red (Km^R^), which chromosomally encodes a λ red recombinase whose expression is induced by arabinose (Hashimoto and Kato, 2003). The mutant plasmid (pEC41Δ*fec*; Table 1) was constructed in MG1655red (Km^R^) using λ red recombinase-mediated recombination to replace the *fec* system (*fecIRABCDE*) with a chloramphenicol resistance cassette. Then, pEC41Δ*fec* was transferred from the strain to other bacterial hosts through conjugation or transformation by electroporation.

To construct Δ*fec*^pEC41^-EC41, the pEC41Δ*fec* plasmid was conjugatively transferred into EC41 from MP10/pEC41Δ*fec* (Table 1). The resulting transconjugant that harbored both pEC41 and pEC41Δ*fec* was cultured in LB medium containing chloramphenicol to select for the presence of pEC41Δ*fec* and to facilitate the expulsion of pEC41 from the bacteria. After 12 h of culture, the bacteria were spread on LB agar supplemented with chloramphenicol. Δ*fec*^pEC41^-EC41 that harbored pEC41Δ*fec* but not pEC41 was confirmed using PCR to detect the size of the *fec* locus in the plasmid.

### Plasmid Profile Analysis

The method reported by Kado and Liu (1981) was used to extract plasmid DNA and determine the plasmid profiles in *E. coli* strains, with some modifications. Briefly, one milliliter of an overnight bacterial culture was pelleted by centrifugation at 14,000 × *g* for 5 min at 4°C. The resulting bacterial pellet was resuspended in 60 μl of preheated lysis buffer (1.5% sodium dodecyl sulfate and 0.2 N NaOH, pH 12.8) to lyse bacterial cells, followed by mixing with an equal amount of PCI (phenol:chloroform:isoamyl alcohol 25:24:1). After centrifugation, 20 μl of the aqueous phase (the upper layer of the solution) was mixed with 4 μl of DNA dye and subjected to electrophoresis in a 0.6% agarose gel.

### Iron Growth Promotion Assay

The ability of *E. coli* strains to utilize various iron sources was determined by performing an iron growth promotion assay. Overnight cultures (1 ml) of the transconjugants or the control strains were centrifuged, washed with PBS, and then resuspended in 10 ml of PBS. The bacterial suspension (100 μl) was spread onto 15 ml of iron-limited medium (ILM) agar composed of 15% agar and ILM medium [0.1 mM CaCl_2_, 50 mM Na_2_HPO_4_, 20 mM KH_2_PO_4_, 10 mM NaCl, 20 mM NH_4_Cl, 20% glucose, 2 mM MgSO_4_, 0.025 μM sodium citrate, and 100 μM 2,2′-bipyridyl (DIP)]. The resulting bacterium-containing agar plates were punched with pipette tips to create wells of approximately 4-mm diameter, and 10 μl of ferrous sulfate (10 mM), ferric chloride (10 mM), or ferric citrate (10 mM) were added to the wells as iron sources for the bacteria in the agar. The plates were incubated at 37°C for 24 h, and the diameter of growing bacteria surrounding each well was measured.

### Plasmid Stability Assays Using pEC41

Because pEC41 conferred ampicillin resistance, the *E. coli* strains harboring pEC41 were cultured in LB medium with ampicillin (50 μg/ml) overnight. Then the cultures were transferred to fresh ampicillin-containing (50 μg/ml) LB medium in a 1:100 ratio and incubated for 2 h to ensure that all the live cells harbored the plasmid. Then, the bacteria were transferred to fresh LB medium in a 1:100 ratio. The bacterial cultures were spread on LB agar at different time points. The percentages of pEC41-harboring bacteria were determined by randomly selecting 50 colonies and determining their abilities to grow on LB agar containing ampicillin.

### Plasmid DNA Extraction and Sequencing Analysis

The pEC41 plasmid was purified from MP10/pEC41 (Table 1) by using a QIAGEN Large Construction Kit (QIAGEN, United Kingdom, Cat. no. 12462) and subjected to sequencing by using the Illumina MiSeq Platform in a paired-end configuration. The sequencing data were *de novo* assembled into contigs using CLC Genomic Workbench. The gaps in the contigs were closed by Sanger sequencing the PCR fragment amplified between the contigs. The primers used to close the gaps are shown in Supplementary Table 1. Proteins were predicted using SnapGene and NCBI CD searches. A similarity search and pairwise alignment were performed using BLAST programs. The sequences of pEC41 are deposited in the GenBank repository, accession number MW548582.

### Mouse Infection Models

For the mouse systemic infection models, the *E. coli* strains EC41 and Δ*fec*^pEC41^-EC41 (1 × 10^7^ CFU/per strain) were coinoculated or independently inoculated into 6–7-week-old BALB/c mice through a tail vein injection as described previously (Subashchandrabose et al., 2013), with some modification. At 24 h postinfection (hpi), blood, hearts, livers, spleens, kidneys and lungs were collected. The solid tissue samples were weighed and homogenized in 3 ml of 0.85% NaCl. The bacterial counts in the blood and the homogenates were determined by plating the samples on LB agar containing appropriate antimicrobials. For the coinfection experiments, EC41 and Δ*fec*^pEC41^-EC41 were differentiated by chloramphenicol resistance (Table 1).

For the UTI models, 8–10-week-old female C3H/HeN mice were utilized. Equal numbers (1 × 10^8^ CFU) of the strains harboring pEC41 or pEC41Δ*fec* were coinoculated into the animals transurethrally as described previously (Wright et al., 2005), with some modification. At 24 hpi, the mice were sacrificed, and the bladders and kidneys were collected, weighed, and homogenized. The organ bacterial counts were then determined and differentiated by chloramphenicol resistance, because pEC41Δ*fec*, which harbored a chloramphenicol resistance cassette, conferred chloramphenicol resistance to host bacteria, while pEC41 didn’t (Table 1). Equal volumes of the tissue homogenates were plated on LB agar without and with chloramphenicol (15 μl/ml), respectively. The colony numbers on the LB agar reflected the total bacterial counts in the samples, while the numbers on the chloramphenicol-containing LB agar reflected the counts of pEC41Δ*fec*-harboring strains. The counts of pEC41-harboring strains were determined by subtracting the total bacterial counts by the pEC41Δ*fec*-harboring strain counts in the samples.

The competitive indices (CIs) were calculated by dividing the ratio of pEC41Δ*fec*-harboring to the pEC41-harboring strains in the output by the ratio of the two strains in the inoculum [(CFU_pEC41_Δ*_fec_*/CFU_pEC41_)_output_/(CFU_pEC41_Δ*_fec_*/CFU_pEC41_) _input_]. All experimental procedures were approved by the Institutional Animal Care and Use Committee (IACUC) of National Cheng Kung University, Tainan City, Taiwan (approval no. 108130).

### Serum and Urine Survival Assay

For serum survival assays, equal numbers of bacterial strains harboring pEC41 and pEC41Δ*fec* (2 × 10^5^ CFU/per strain) were coincubated with 40 μl of 90% heat-inactivated pooled mouse serum (5 mice) in PBS at 37°C. After different incubation periods, the live bacterial counts of the strains were determined by spreading the culture on LB agar plates. The strains harboring pEC41 and pEC41Δ*fec* were differentiated by chloramphenicol resistance, which was conferred by pEC41Δ*fec* but not pEC41. Equal volumes of the serum samples were plated on LB agar without and with chloramphenicol (15 μl/ml), respectively. The colony numbers on the LB agar reflected the total bacterial counts in the samples, while the numbers on the chloramphenicol-containing LB agar reflected the counts of pEC41Δ*fec*-harboring strains. The counts of pEC41-harboring strains were determined by subtracting the total bacterial counts by the pEC41Δ*fec*-harboring strain counts in the samples.

As for urine survival assay, equal numbers of bacterial strains harboring pEC41 and pEC41Δ*fec* (4 × 10^3^ CFU/per strain) were coinoculated in 80 μl of 90% pooled human urine (5 donners) in PBS at 37°C for 4 h and the live bacterial counts of the strains were determined and differentiated by chloramphenicol resistance. Then, the bacterial culture were transferred to fresh 90% urine in a 1:250 dilution for every 4 h of incubation at 37°C and the live bacterial counts were determine right before each transfer.

### RNA Isolation and Real-Time PCR

Bacteria were grown in M9 medium containing 100 μM DIP with and without supplementation of 20 μM ferric citrate for 16 h at 37°C with shaking at 200 rpm. RNA was extracted from bacteria using the TRI Reagent according to the manufacturer’s instructions (Sigma-Aldrich, St. Louis, MO, United States). To remove contaminating DNA, the resulting RNA was treated with DNase I (Roche Applied Science, Mannheim, Germany) at 37°C for 1.5 h. Then, the mixture was subjected to phenol/chloroform (1:1) extraction and ethanol precipitation. Finally, the purified RNA was dissolved in RNase-free water and stored at −80°C. The complementary DNA (cDNA) was converted from the purified RNA using random hexamer and Moloney Murine Leukemia Virus (M-MLV) reverse transcriptase according to manufacturer’s instruction (Invitrogen, Carlsbad, CA, United States). For real-time PCR, the cDNA was mixed with primers and KAPA SYBR FAST qPCR Master Mix (Kapa Biosystems, Boston, MA, United States), followed by PCR using StepOnePlus Real-Time PCR Systems with standard reaction conditions (Applied Biosystems, Carlsbad, CA, United States). The primers used in the assays were shown in Supplementary Table 1. The expression level of *fecA* was normalized to that of *ftsZ*.

### Distribution of *fecA*

The frequencies of *fecA* among *E. coli* were determined by PCR-based analysis. The primers used in this analysis were shown in Supplementary Table 1. The PCR analyses were carried out in a 10 μl volume consisting 1.1 μl of MQ water, 1 μl of 10× Taq Buffer, 0.8 μl dNTP (2.5 mM), 5 μl of template DNA, 1 μl of each primer (20 μM), and 0.1 μl of Taq polymerase. Taq PCR condition was as follows: an initial denaturing step at 95°C for 5 min, followed by 30 cycles of 95°C of denaturation for 1 min, annealing at 55°C for 30 s, and elongation at 72°C for 1 min, and final extension at 72°C for 10 min. The PCR products were separated by gel electrophoresis on 1% agarose gel [1 g agarose in 100 ml of 1× Tris-Acetic acid-EDTA (TAE) buffer]. Agarose was dissolved by heating and cooled down before adding ethidium bromide (EtBr) to a final concentration of approximately 0.05 μg/ml. 2.5 μl of sample was typically loaded to each well along with molecular weight DNA ladder. The DNA fragments were separated by electrophoresis at 100 V for about 30 min and were visualized under UV light.

### Statistical Analysis

Before statistical analysis, each set of the experiment data was tested for the normality using the Shapiro–Wilk test. The data whose normality was rejected by the test (*P* < 0.05) were subsequently subjected to non-parameteic statistical analyses. Otherwise, the data (*P* ≥ 0.05 in the normality test) were subjected to parametric statistical analyses. For non-parametric analyses, the Mann–Whitney *U*, Wilcoxon signed-rank, and Wilcoxon rank-sum tests were utilized to analyze the results of mouse independent infection, mouse coinfection, and some iron growth promotion assays, respectively. For the parametric analyses, the one-way ANOVA was utilized to analyze the results of plasmid stability, real-time PCR, and some iron growth promotion assays. The two-way ANOVA was utilized to analyze independent culture experiments. The paired two-tailed student’s *t*-test was used to analyze the results of bacterial growth in co-culture experiments. Comparisons involving the frequencies of *fecA* in different source groups of *E. coli* isolates were analyzed by the two-tailed Fisher’s exact test. A *P*-value of <0.05 was arbitrarily set as the threshold for statistical significance.

## Results

### The *E. coli* Plasmid pEC41 Provides Iron Uptake-Deficient Strains the Ability to Take Up Iron

**FIGURE 1:**
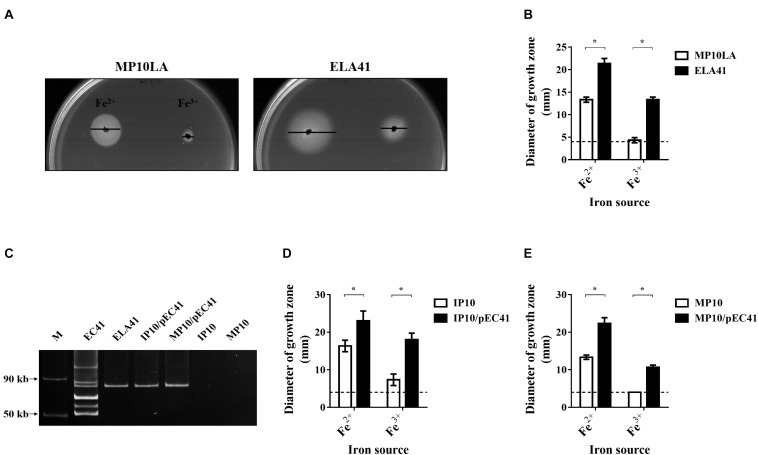
The growth of the transconjugant ELA41 under iron-limited conditions and the plasmid the transconjugant obtained through conjugation. **(A)** Growth promotion assays of ELA41 and MP10LA with Fe^2+^ (ferrous sulfate) and Fe^3+^ (ferric chloride). Iron-limited medium (ILM) agar was seeded with ELA41 and MP10LA. Wells with a diameter of 4 mm were punched into agar plates and filled with 10 μl of 10 mM ferrous sulfate and ferric chloride. The diameters of growth zones around the wells were determined after 24 h incubation at 37°C. Bars indicate the diameters of the growth zones. **(B)** Quantification of the diameters of the growth zones of ELA41 and MP10LA. Dashed lines indicate the diameter of the wells (4 mm) containing iron sources. The experiments were performed in triplicate and presented as the means ± SD. **(C)** The plasmid profiles of EC41, ELA41, IP10/pEC41, and MP10/pEC41. The plasmids were extracted using the method describe by Kado and Liu (1981). The plasmid extracts without any restriction enzyme digestion were subjected to agarose gel electrophoresis. Therefore, the plasmids in the gel were in a circular form. M: Fifty- and 90-kb plasmid markers. The markers were extracted from *Salmonella choleraesuis* OU7526 (Tzeng et al., 2012). **(D,E)** Quantitative analysis of the growth promotion assays of IP10 **(D)** and MP10 **(E)** strains with and without pEC41. The experiments were performed in triplicate and are presented as the means ± SD. *P < 0.05 (Wilcoxon rank-sum test).

To screen for antimicrobial-resistant conjugative plasmids that provide iron uptake ability, an *E. coli* mutant defective in iron uptake was constructed and utilized to collect ampicillin-resistant conjugative plasmid through conjugation. The mutant strain was an *E. coli* MG1655 strain with an *entA* deletion (MP10LA; Table 1). Because the *entA* gene is essential for enterobactin synthesis (Liu et al., 1989), MP10LA lacked the enterobactin-dependent iron uptake system and thus exhibited growth defects in iron-deficient medium. MP10LA served as the recipient to capture ampicillin-resistant plasmids through conjugation from 50 donor *E. coli* clinical isolates that were associated with bacteremia or UTIs. Thirty-six transconjugants were obtained from the conjugations. The plasmids that were able to promote the growth MP10LA under iron-limited conditions are likely to harbor iron uptake-related genes. Thus, these transconjugants were subjected to growth promotion assays in iron-limited medium (ILM; see section “Materials and Methods”) agar. Among them, the transconjugant ELA41 (Table 1) showed an increased iron uptake ability (Figures 1A,B). We further investigated the plasmid patterns of ELA41 and its corresponding donor clinical isolate EC41. The plasmids were extracted from the strains using the method described previously (Kado and Liu, 1981) (please check section “Materials and Methods”) and subjected to agarose gel electrophoresis. As shown in Figure 1C, the plasmid extract of ELA41 exhibited one band with a size ranging from 50 to 90 kb on the gel, while that of EC41 showed multiple bands. The band in ELA41 showed a size similar to that of one of the bands in EC41. These findings suggest that the transconjugant ELA41 acquired one plasmid from EC41. The plasmid was named pEC41. The multiple bands in EC41 suggest that the isolate harbor multiple plasmids. However, some of the bands may be resulted from the fragmentation of plasmid DNA at the time of extraction.

Two sequential conjugation experiments were performed to determine whether pEC41 and the increased iron uptake ability in ELA41 were able to be conjugatively transferred to other bacterial hosts. First, ELA41 served as the donor strain, and an MG1655 *entA* mutant-derived strain, IP10 (Table 1), served as the recipient strain. The resulting transconjugant was designated IP10/pEC41. Then, IP10/pEC41 served as the donor strain, and another MG1655 *entA* mutant-derived strain, MP10 (Table 1), served as the recipient strain. The resulting transconjugant was MP10/pEC41. Similar to ELA41, IP10/pEC41, and MP10/pEC41 harbored pEC41 (Figure 1C), and these transconjugant showed increased iron uptake abilities in the growth promotion assay compared to their corresponding recipient strains (Figures 1D,E). These findings suggest that pEC41 can be transferred though conjugation independent of its original host *E. coli* (EC41) background and that the iron uptake ability is transferred with the plasmid.

### The pEC41 Plasmid Belongs to the IncM Plasmid Groups That Have Been Shown to Carry Multiple Antimicrobial Resistance Genes, Including ESBL-Encoding Genes

**FIGURE 2:**
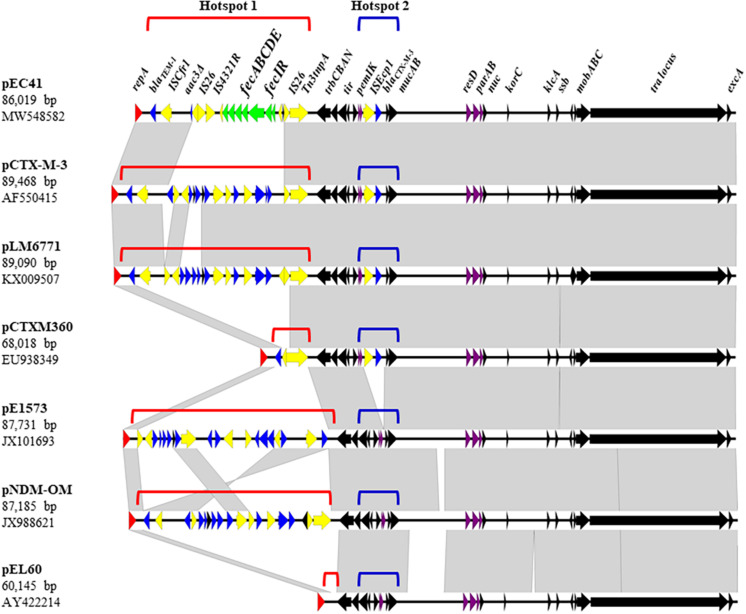
Linear comparison of pEC41 with other IncM group plasmids. Black arrows indicate the common regions shared among the plasmids. The *tra* locus contains *tra* genes involved in conjugal transfer. The partition system (*parAB*) and postsegregational killing system (*pemIK*) are indicated by purple arrows. Resistance genes are indicated by blue arrows. Insertion sequences and transposon components are indicated by yellow arrows. The *fec* system on pEC41 is indicated with green arrows. The replicase gene *repA* is indicated by red arrows. The direction of arrows indicates the direction of the ORF. Gray shading denotes regions of shared homology among different plasmids. Diagrams were drawn from sequences available under the following GenBank accession numbers: pEC41 (MW548582), pCTX-M-3 (AF550415), pLM6771 (KX009507), pCTXM360 (EU938349), pEl1573 (JX101693), pNDM-OM (JX988621), and pEL60 (AY422214). The comparison map was generated using Easyfig software (Sullivan et al., 2011).

The pEC41 plasmid was purified from ELA41 and subjected to DNA sequencing. This plasmid was 86,019 bp in size with an average G + C content of 52.37%. It harbored 112 potential ORFs, which included a complete array of genes involved in plasmid replication, conjugation and stability, along with a number of mobile genetic elements (Figure 2, Supplementary Table 2, and Supplementary Figure 1). Based on an *in silico* analysis using the web tool PlasmidFinder-2.1^1^ (Carattoli et al., 2014), the pEC41 plasmid belongs to the IncM plasmid groups. Based on BLAST analysis, the backbone structure of pEC41 was highly similar to the backbone structures of a group of multiple antimicrobial resistance genes (ARGs)-bearing IncM plasmids (Carattoli et al., 2015), such as pCTX-M3 from *Citrobacter freundii* (85% query coverage; 99% identity) (Golebiewski et al., 2007), pLM6771 from *E. coli* (84% query coverage; 99% identity) (Gong et al., 2019), pCTXM360 from *Klebsiella. pneumoniae* (77% query coverage; 99% identity) (Zhu et al., 2009), pEl1573 from *Enterobacter cloacae* (80% query coverage; 99% identity) (Partridge et al., 2012), and pNDM-OM from *K. pneumoniae* (81% query coverage; 99% identity) (Bonnin et al., 2013). The most conserved parts among these plasmids were the regions encoding the transfer loci (*tra*), the replicon function (*repA*), the partitioning factors (*parA* and *parB*), the postsegregational killing system (*pemI* and *pemK*), and a large number of hypothetical proteins (Figure 2, Supplementary Table 2, and Supplementary Figure 1) (Golebiewski et al., 2007). These plasmids exhibited two insertion hotspots (Hotspot 1 and Hotspot 2) that contained different acquired sequences. These plasmids have been proposed to have evolved from the *Erwinia amylovora* plasmid pEL60, which harbors no antimicrobial resistance gene and no insertion sequence in the hot spots (Bonnin et al., 2013) (Figure 2).

Hotspot 1 was located between the replication locus (*rep*) and the Trb transfer operon (*trbCBAN*) (Figure 2). In pEC41, a *fec* system (*fecIRABCDE*) was identified in this locus, which may be responsible for the iron uptake ability. The *fec* system encoded in this plasmid was designated *fec*^pEC41^ in this study. In pCTXM360, a Tn*3* transposon that carries the broad-spectrum β-lactamase-encoding gene *bla*_TEM–1_ (Ghafourian et al., 2015) was located in the hotspot. In pEC41, pCTX-M3, pLM6771, and pNDM-OM, IS-mediated insertions (IS*26* and/or ISC*fr1*) occurred in Tn*3*, leading to the truncation of a part of the Tn*3* structure with the *bla*_TEM–1_ genes remaining in the loci. The IS insertions brought extra antimicrobial resistance genes into pCTX-M3, pLM6771, and pNDM-OM and brought *fec*^pEC41^ into pEC41.

Hotspot 2 was located between *pemIK* and the UV resistance genes (*mucAB*), in which the insertion sequence IS*Ecp1*with the ESBL-encoding gene *bla*_CTX–M–3_ (Golebiewski et al., 2007) was inserted in four of the six multiple ARGs-bearing plasmids (Figure 2). The mobilization of *bla*_CTX–M–3_ gene may occur with the assistance of the IS*Ecp1* element, which were commonly found 127 bp upstream of the antimicrobial resistance gene (Literacka et al., 2009; Zong et al., 2010). On the other hand, pEl1573 and pNDM-OM lacked the IS*Ecp1-bla*_CTX–M–3_ sequence at this locus.

Taken together, the plasmid pEC41 carried *fec*^pEC41^ and *bla*_TEM–1_ in the hot spot 1 and harbored *bla*_CTX–M–3_ in the hot spot 2.

### The *fec* System in pEC41 Enhances the Iron Uptake Ability of Iron Uptake-Deficient Strains

**FIGURE 3:**
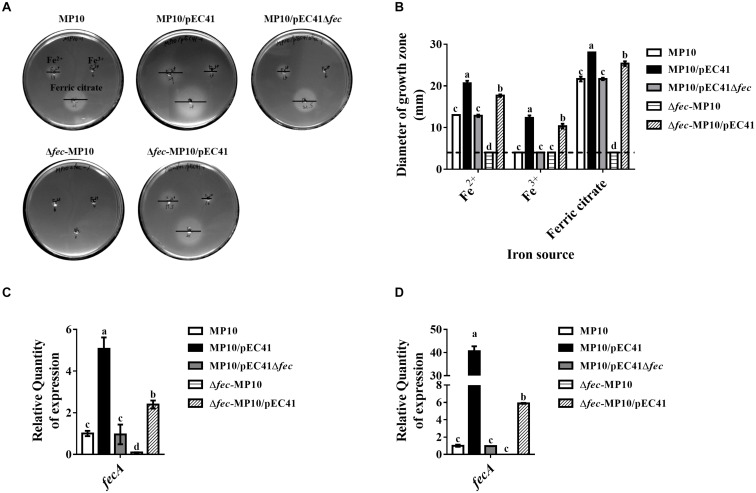
The *fec* system of pEC41 provides iron uptake abilities. **(A)** Iron growth promotion assays of MP10 strains harboring pEC41 or pEC41Δ*fec* with and without the chromosomal deletion of *fec*^MG1655^. ILM agar plates were seeded with the indicated strains, and three punched wells on each plate were loaded with 10 μl of 10 mM ferrous sulfate (Fe^2+^; top left panel), ferric chloride (Fe^3+^; top right panel) and ferric citrate (bottom panel). The diameters of growth zones around the wells were determined after 24 h of incubation at 37°C. Bars indicated the diameters of the growth zones. **(B)** Quantification of the diameters of the growth zones of the strains in **(A)**. Dashed lines indicate the diameter of the wells (4 mm) of iron sources. **(C,D)** The mRNA levels of *fecA* in strains grown under iron-limited conditions without or with supplementation with ferric citrate. The bacteria were grown in M9 medium containing 100 μM DIP without **(C)** and with **(D)** supplementation of 20 μM ferric citrate overnight at 37°C. Levels of the *fecA* transcript were determined with real-time PCR using primers able to recognize the *fecA* genes in *fec*^pEC41^ and *fec*^MG1655^. The *fecA* transcript level in each strain was normalized to the *ftsZ* level and presented as the relative level compared to that of MP10. The experiments were performed in triplicate and presented as the means ± SD. Different letters marked above the bars indicate groups with significant differences (*P* < 0.05, one-way ANOVA).

The *fec*^pEC41^ sequence was deleted in pEC41 (pEC41Δ*fec*) and the effect of the deletion on iron uptake by bacteria was evaluated to investigate whether *fec*^pEC41^ is responsible for conferring the iron uptake ability. Since MG1655 and its derived strains chromosomally encode a copy of the *fec* system that shares 94% identity with the *fec*^pEC41^ system (data not shown), the chromosomal copy of the *fec* system in the MG1655-derived strains was designated as *fec*^MG1655^ to differentiate it from *fec*^pEC41^. The *E. coli* strains MP10, MP10/pEC41, MP10/pEC41Δ*fec*, Δ*fec*-MP10/pEC41, and Δ*fec*-MP10/pEC41Δ*fec* (Table 1), which harbored *fec*^MG1655^, *fec*^pEC41^/*fec*^MG1655^, *fec*^MG1655^, *fec*^pEC41^, and no *fec* system, respectively, were evaluated for their abilities to utilize ferrous sulfate (Fe^2+^), ferric chloride (Fe^3+^) and ferric citrate. MP10/pEC41 exhibited larger growth zones around the iron sources than MP10/pEC41Δ*fec* (Figures 3A,B), indicating that *fec*^pEC41^ is responsible for the iron uptake ability encoded in pEC41. MP10/pEC41, which harbored 2 copies of the *fec* system, exhibited the largest growth zones around the iron sources, while the strains harboring one copy of the *fec* system (MP10, MP10/pEC41Δ*fec*, and Δ*fec*-MP10/pEC41) showed larger growth zones around ferrous sulfate and ferric citrate than the strain without any *fec* system (Δ*fec*-MP10; Table 1). Based on these results, *fec*^MG1655^ and *fec*^pEC41^ exert additive effects on the iron uptake ability.

We investigated whether the expression levels of the *fecABCDE* operons in these strains were correlated with their iron uptake abilities. Under iron-depleted conditions (M9 medium with 100 μM DIP) with or without ferric citrate, the total *fecA* mRNA levels, including the mRNA derived from *fec*^pEC41^ and *fec*^MG1655^, in MP10/pEC41 were significantly higher than those in the strains harboring either *fec*^MG1655^ or *fec*^pEC41^ (Figures 3C,D). The Δ*fec*-MP10/pEC41 strain showed higher levels of expression than MP10/pEC41Δ*fec* and MP10, suggesting that *fec*^pEC41^ may provide a higher level of *fecA* expression than *fec*^MG1655^. In addition, supplementation with ferric citrate significantly increased *fecA* expression, especially in MP10/pEC41 cells (Figures 3C,D). Therefore, the expression of the *fecABCDE* genes is consistent with the iron uptake abilities of these strains.

In addition, to investigate whether *fec*^pEC41^ contributes to EC41 survival in ILM, the *fec*^pEC41^ deletion mutant of EC41 (Δ*fec*^pEC41^-EC41) was constructed by conjugatively transferring pEC41Δ*fec* into EC41 to compete with and thus replace pEC41 (Table 1). Then, the EC41 strains with and without *fec*^pEC41^ were subjected to iron growth promotion assays. Unlike the MP10 strains, both EC41 and Δ*fec*^pEC41^-EC41 grew in ILM agar without additional iron supplementation (data not shown), suggesting that *fec*^pEC41^ may not play a major role in EC41 growth in ILM.

### The pEC41 Plasmid Is Stable in EC41, MP10, and the Cystitis-Associated Isolate A487, but Not the UPEC Strains CFT073^str^ and UTI89^str^

**FIGURE 4:**
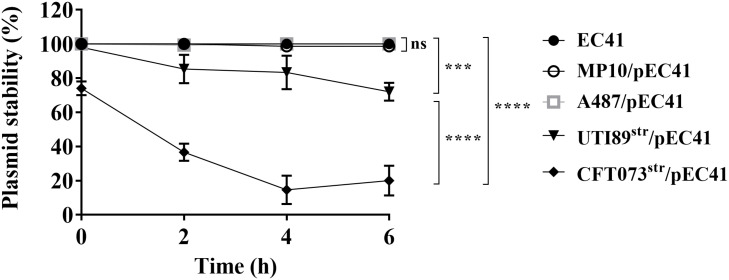
The stability of pEC41 in different *E. coli* host strains. The stability of pEC41 in EC41, MP10, A487, UTI89^str^, and CFT073^str^ was evaluated. The percentage of pEC41-containing cells was determined by measuring the percentage of ampicillin-resistant cells at the indicated time points. The experiments were performed in triplicate and are presented as the means ± SD. ***P < 0.001, ****P < 0.0001; ns, no significant difference (one-way ANOVA).

To determine whether pEC41 is stably maintained in different *E. coli* strains, the stabilities of the plasmid in EC41, MP10, the prototype UPEC strains UTI89 and CFT073 (Welch et al., 2002; Chen et al., 2006), and a cystitis-associated clinical isolate, A487 (Table 1), were assessed. A487 acquired pEC41 through transformation to become A487/pEC41. CFT073 and UTI89 encode no known antimicrobial resistance genes as markers for conjugation experiments, we utilized spontaneous streptomycin-resistant mutants of the strains (CFT073^str^ and UTI89^str^; Table 1) to conjugatively acquire pEC41 from EC41, resulting in CFT073^str^/pEC41 and UTI89^str^/pEC41 (Table 1). After an incubation in LB medium for different periods, the percentages of pEC41-harboring cells in EC41, MP10/pEC41, A487/pEC41, CFT073^str^/pEC41, and UTI89^str^/pEC41 cultures were determined. As shown in Figure 4, pEC41 was stably maintained in MP10 and the clinical *E. coli* isolates EC41 and A487. However, the percentages of pEC41-containing cells in CFT073^str^/pEC41 and UTI89^str^/pEC41 cultures decreased significantly during incubation. These findings suggest that the stability of pEC41 depends on its host backgrounds. In addition, since CFT073 harbors no plasmid and UTI89 harbors an IncFIB/IIA plasmid that is not in the same incompatibility group as pEC41 (Welch et al., 2002; Chen et al., 2006), the instability of pEC41 in the strains was not likely due to plasmid interference from the bacterial hosts.

### *fec*^pEC41^ Contributes to the Virulence of EC41 in Systemic Infections

**FIGURE 5:**
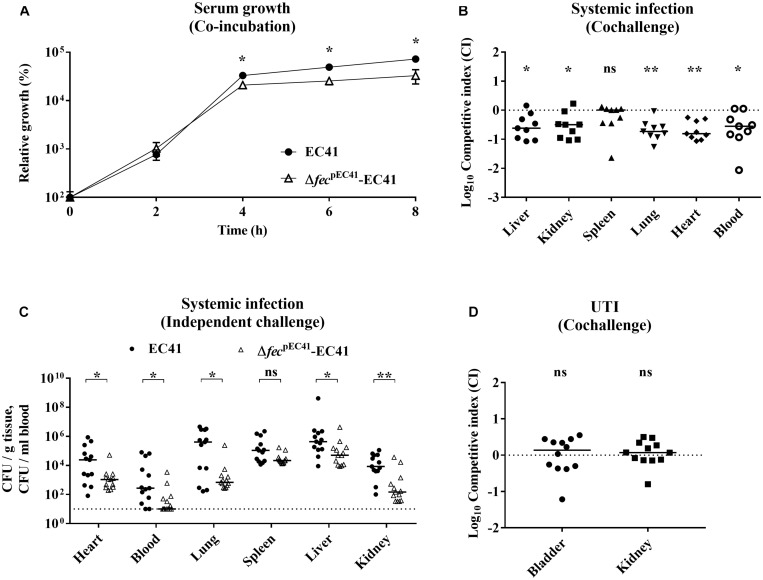
The roles of fec^pEC41^ in EC41 growth in heat-inactivated serum and in the pathogenesis of systemic infections and urinary tract infections. **(A)** The growth of EC41 and Δ*fec*^pEC41^-EC41 cocultured in 90% heat-inactivated mouse serum. The results are presented as the means ± SD. The data are shown as the relative growth rates compared to the bacterial counts of the original inoculums. The data are representative of three independent experiments performed in triplicate. **(B)** Cochallenge of EC41 and Δ*fec*^pEC41^-EC41 in a mouse model of systemic infection. Equal numbers of EC41 and Δ*fec*^pEC41^-EC41 were coinoculated into BALB/c mice via the tail vein (*N* = 9). At 24 h postinfection, the bacterial numbers of each strain in the liver, kidney, spleen, lung, heart, and blood were determined, and the competitive indices (CI) of Δ*fec*^pEC41^-EC41 vs. EC41 in these tissues were calculated. **(C)** Independent challenge with EC41 and Δ*fec*^pEC41^-EC41 in the mouse model of systemic infection. The bacterial counts in the organs and blood were determined at 24 h postinfection. (*N* = 13 for the animals infected with each bacterial strain). **(D)** Cochallenge of EC41 and Δ*fec*^pEC41^-EC41 in a mouse model of UTIs. Equal numbers of the two strains were transurethrally coinoculated into female C3H/HeN mice (*N* = 12). At 24 h postinfection, the bacterial counts and the CI of Δ*fec*^pEC41^-EC41 vs. EC41 in bladders and kidneys were determined. The horizontal bars indicate median values. For **(C)**, the dashed line represents the detection limit. For **(B,D)**, the dashed lines represent the log_10_ scale of CI = 1. The CI in the cochallenge experiments was calculated using bacterial counts with the equation [(Δ*fec*^pEC41^-EC41 CFU/EC41 CFU)_output_/(Δ*fec*^pEC41^-EC41 CFU/Δ*fec*^pEC41^-EC41 CFU)_input_]. The log_10_ scale of CI values less than 0 (CI = 1) indicates a comparative defect in Δ*fec*^pEC41^-EC41. The paired two-tailed student’s *t* test and The Mann–Whitney *U* test was used for the statistical analyses of **(A,C)**. The Wilcoxon signed-rank test was used in **(B,D)**. *P < 0.05, **P < 0.01; ns, no significant difference.

EC41, which was assigned to sequence type (ST) 405, phylogroup D, and *fimH*27, was isolated from the bloodstream of a patient, suggesting that this pathogen causes systemic infections. We evaluated whether *fec*^pEC41^ contributes to EC41 survival in serum, in which the bioavailability of iron is restricted (Hood and Skaar, 2012). In coincubation experiments, EC41 outcompeted Δ*fec*^pEC41^-EC41 in heat-inactivated serum (Figure 5A). Based on these results, *fec*^pEC41^ confers a fitness advantage in terms of the serum survival of EC41 and *fec*^pEC41^ may contribute to the pathogenesis of *E. coli*.

The ability of Δ*fec*^pEC41^-EC41 to cause systemic infection was compared to that of EC41 to further determine whether *fec*^pEC41^ facilitates *E. coli* infection. In cochallenge experiments, equal numbers of EC41 and Δ*fec*^pEC41^-EC41 were intravenously coinoculated into mice. As shown in Figure 5B, at 24 h postinfection, Δ*fec*^pEC41^-EC41 was outcompeted by EC41 in the liver, kidneys, lungs, heart, and blood. Similarly, in independent challenge experiments, mice infected with EC41 showed significantly higher bacterial burdens in the organs and blood than those infected with Δ*fec*^pEC41^-EC41 (Figure 5C). Thus, *fec*^pEC41^ contributes to the pathogenesis of EC41 in systemic infections.

In addition, we examined whether *fec*^pEC41^ contributed to UTIs. EC41 and Δ*fec*^pEC41^-EC41 were intraurethrally coinoculated into mice. However, similar bacterial counts of the wild-type and mutant strains were observed in the bladders and kidneys at 24 h postinfection (Figure 5D), suggesting that *fec*^pEC41^ may not play a significant role in EC41-mediated UTIs.

### *fec*^pEC41^ Promotes the Fitness of A487 During Urinary Tract Infections

**FIGURE 6:**
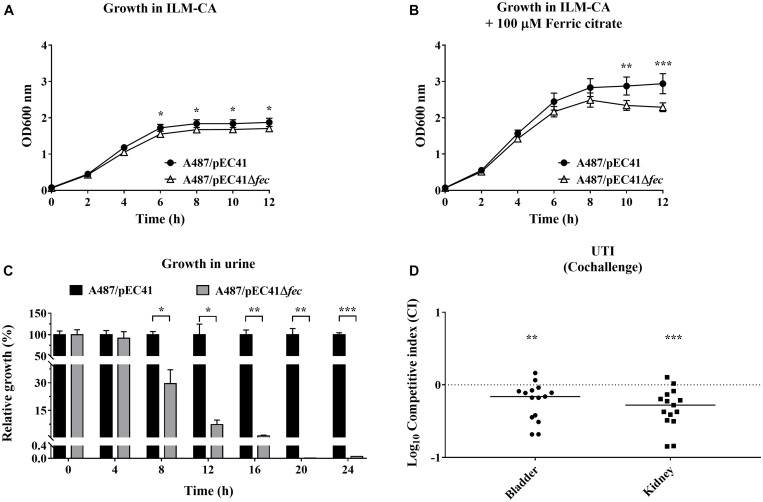
The effect of *fec*^pEC41^ on the growth of A487 under iron-limited conditions and in urine and on the ability of the bacteria to colonize urinary tracts. **(A)** The growth of A487/pEC41 and A487/pEC41Δ*fec* cultured in ILM-CA. **(B)** The growth of A487/pEC41 and A487/pEC41Δ*fec* cultured in ILM-CA supplemented with 100 μM ferric citrate. **(C)** The growth of A487/pEC41 and A487/pEC41Δ*fec* cocultured in urine. The results are shown as the relative growth rates compared to A487/pEC41 at each indicated time point. For **(A–C)**, the results are presented as the means ± SD. The data are representative of three independent experiments performed in triplicate. **(D)** Cochallenge of A487/pEC41 and A487/pEC41Δ*fec* in a mouse model of UTI. Equal numbers of the two strains were transurethrally coinoculated into female C3H/HeN mice (*N* = 15). At 24 h postinfection, the bacterial counts and the CI of A487/pEC41Δ*fec* vs. A487/pEC41 in bladders and kidneys were determined. The horizontal bars indicate median values. The dashed line represents the log_10_ scale of CI = 1. The two-way ANOVA was used in the statistical analysis of **(A,B)**. The paired two-tailed student’s *t*-test was used in the statistical analysis of **(C)**, while the Wilcoxon signed-rank test was used in **(D)**. *P < 0.05, **P < 0.01, ***P < 0.001.

pEC41 could be stably maintained by A487 cells. We further investigated whether *fec*^pEC41^ contributes to the host’s abilities to grow in an iron-limited condition and to cause infection when A487 obtains pEC41. The growth of A487/pEC41was compared with that of A487/pEC41Δ*fec*. Since these strains were unable to grow on ILM agar with and without iron supplementation (data not shown), we measured their growth in ILM broth supplemented with 1% casamino acid as a carbon source (ILM-CA). A487/pEC41 showed higher levels of growth than A487/pEC41Δ*fec* in ILM-CA and in ILM-CA supplemented with ferric citrate (Figures 6A,B). Supplementation with ferric citrate increased the difference in growth between the strains with and without *fec*^pEC41^. These findings suggested that *fec*^pEC41^ significantly contributes to A487 growth under iron-limited conditions.

Because A487 was associated with UTIs, the growth of A487/pEC41 and A487/pEC41Δ*fec* in the urine was evaluated. Equal amounts of A487/pEC41 and A487/pEC41Δ*fec* were coinoculated in 90% human urine and incubated at 37°C. At intervals of 4 h, the bacterial counts of the two strains were determined, and the cultures were transferred to fresh 90% urine in a 1:250 dilution for the next 4 h of incubation. As shown in Figure 6C, A487/pEC41 significantly outcompeted A487/pEC41Δ*fec* after 12 h of incubation in urine. Based on these findings, the *fec* system optimizes the fitness of A487 growing in urine.

Furthermore, we investigated whether *fec*^pEC41^ facilitates A487 colonization of the urinary tract. A487/pEC41 and A487/pEC41Δ*fec* were coinoculated into the urinary tract of mice. At 24 h postinfection, the bacterial loads of A487/pEC41 outcompeted those of A487/pEC41Δ*fec* in bladders and kidneys (Figure 6D), suggesting that *fec*^pEC41^ increases the fitness of A487 to infect urinary tracts.

### The Distribution of the *fec* System in Fecal and Clinical *E. coli* Isolates

Because the *fec* system increased the virulence or fitness of *E. coli* to cause infections (Figures 5C, 6D), we scrutinized whether the distribution of the system was associated with *E. coli* infections. We determined the distribution of the *fec* system by performing PCR analysis with primers that probe the *fecA* gene (the first gene in the *fecABCDE* operon of the *fec* system). The primers were designed to target the conserved region of the *fecA* gene. Therefore, the *fecA* alleles of *fec*^pEC41^ and *fec*^MG1655^ were both detected in the PCR-based analysis. The *fecA* frequencies in commensal fecal *E. coli* and UTI- and bacteremia- associated clinical isolates were investigated. As shown in Table 2, the cystitis-, pyelonephritis-, and bacteremia- associated *E. coli* groups showed significantly higher frequencies of *fecA* than the fecal isolates. These findings suggest that the *fec* system is positively associated with *E. coli* infections in humans and thus suggest their pathogenic roles in bacterial infections in humans.

**TABLE 2:** Distribution of *fecA* in different source groups of *E. coli* isolates.

	No. (%) of *E. coli* isolates				*P-*value^a^		
Gene	Fecal isolates (*N* = 87)	Cystitis isolates (*N* = 67)	Pyelonephritis isolates (*N* = 72)	Bacteremia isolates (*N* = 43)	Fecal vs. cystitis isolates	Fecal vs. pyelonephritis isolates	Fecal vs. bacteremia isolates
*fecA*	22 (25.29)	37 (55.22)	40 (55.56)	22 (51.62)	<0.001	<0.001	0.005

*^a^Fisher’s exact test.*

## Discussion

The present study identified a novel conjugative plasmid, pEC41, which was able to promote the iron uptake ability of *E. coli*, thus contributing to the pathogenesis of systemic *E. coli* infections and conferring fitness to pathogenic *E. coli* during UTIs. The pEC41 plasmid belongs to the IncM plasmid groups that have been shown to carry multiple ARGs, including ESBL-encoding genes (Figure 2) (Carattoli et al., 2015). To our knowledge, pEC41 is the first IncM group plasmid identified to be able to confer virulence and multiple ARGs (Carattoli et al., 2015). The *fec* system encoded on pEC41 was responsible for promoting the iron uptake ability and virulence of host bacteria. Notably, this study is the first to show that the *fec* iron uptake system contributes to bacterial infections, although it has been previously known to be involved in iron uptake (Braun and Mahren, 2005). Consistent with these findings, our epidemiological investigation showed the association of this system with human bacteremia and UTIs, which supported its role as a bacterial virulence factor in human infections. Thus, the novel plasmid pEC41, which can disseminate virulence and antimicrobial resistance genes though conjugation among bacterial hosts, is a potential threat to public health.

The pEC41 plasmid is highly similar to the well-studied broad-host-range IncM plasmid pCTX-M3, a plasmid isolated from *C. freundii* (Gniadkowski et al., 1998; Baraniak et al., 2002; Golebiewski et al., 2007; Mierzejewska et al., 2007), in which the ESBL-encoding gene *bla*_CTX–M–3_ was originally identified (Golebiewski et al., 2007). pCTX-M3 is a highly conjugative plasmid that can be transferred to solid and liquid media at the same frequency (Golebiewski et al., 2007). This plasmid is proposed to be responsible for the broad dissemination of *bla*_CTX–M–3_ in clinical isolates of *Enterobacteriaceae* in Poland (Baraniak et al., 2002; Golebiewski et al., 2007; Carattoli, 2009). The conjugative transfer system of pCTX-M3 allows plasmid transfer between the soil bacterium *Agrobacterium tumefaciens* and *E. coli* (Golebiewski et al., 2007; Mierzejewska et al., 2007), suggesting that environmental bacteria may serve as a reservoir of these plasmids. The backbone used for the transfer and replication of pEC41 is approximately 100% identical to that of pCTX-M3, suggesting that pEC41 is a plasmid with a broad host range that can effectively transfer among clinically relevant and environmental bacteria. An investigation of the distribution of pEC41 among distinct bacterial pathogens and environmental bacteria would facilitate the evaluation of the plasmid’s threat to public health.

The contribution of the *fec* system to virulence and fitness of *E. coli* is considered niche-dependent and strain-specific because *fec*^pEC41^ facilitated EC41 virulence in systemic infections but not UTIs (Figures 5C,D), while it conferred fitness to A487 in UTIs (Figure 6D). This discrepancy may be because bacterial pathogens always harbor multiple distinct iron uptake systems that may unequally contribute to infections in a specific environment or tissue (functional hierarchy) (Garcia et al., 2011) and because different *E. coli* strains may harbor distinct combinations of iron uptake systems (Welch et al., 2002; Brzuszkiewicz et al., 2006). Iron bioavailability is restricted to invading bacterial pathogens in blood, inner organ tissues, and urinary tracts (Snyder et al., 2004; Skaar, 2010). Although *fec*^pEC41^ plays an important role in systemic EC41 infections, some other iron uptake system(s) in EC41 may play a more dominant role than *fec*^pEC41^ during UTIs, concealing the contribution of the *fec* system. On the other hand, *fec*^pEC41^ may have a relatively higher functional hierarchy in A487 cells and thus confers competitive fitness in the urinary tract.

The finding that the *fec* system contributed to the survival of *E. coli* in the serum and urine suggests that invading pathogenic bacteria may utilize ferric citrate as an iron source in the body. Accordingly, an increase in the availability of ferric citrate in the host may increase the risk of infections caused by bacteria that harbor the *fec* system. The dissemination of the *fec* system would be a potential clinical concern because citrate-based compounds have been utilized as medicines to manage some symptoms associated with bacterial infections. For example, ferric citrate has been utilized to treat hyperphosphatemia in patients with chronic kidney diseases (CKDs) who are easily complicated with UTIs (Naqvi and Collins, 2006; Pennoyer and Bridgeman, 2015). Additionally, citrate compounds have been prescribed as alkalinizers for patients with UTIs to relieve the pain of cystitis (Ueda et al., 2014). Citrate-based medicine may interact with ferric iron and form ferric citrate to serve as an iron source for pathogens in the body. Consequently, treatment with citrate-based medicines may facilitate the survival of invading bacterial pathogens that harbor the *fec* system and thus facilitate the spread of *fec*-encoding plasmids, such as pEC41. The impact of the usage of citrate-based medicine on bacterial infections and the spread of these plasmids must be monitored.

The MP10 strain solely harboring *fec*^pEC41^ (Δ*fec*-MP10/pEC41) exhibited higher levels of iron uptake and *fecA* transcripts than the strains solely harboring *fec*^MG1655^ (MP10 and MP10/pEC41Δ*fec*) (Figure 3). These findings suggest that *fec*^pEC41^ confers a higher level of the *fecA* transcript (i.e., a higher transcript level of the *fecABCDE* operons) and thus contributes a higher level of iron uptake ability than *fec*^MG1655^. Because *fec*^pEC41^ is encoded on pEC41, while *fec*^MG1655^ is located on the chromosome, the copy numbers of pEC41 are likely higher than those of the chromosome, consequently resulting in the presence of multiple copies of *fec*^pEC41^ and a higher level of *fecA* expression in the bacteria. Alternatively, since the sequences of *fec*^pEC41^ and *fec*^MG1655^ are not entirely identical (94% identity), the minor difference in their sequences may cause differences in the promoter activities and/or mRNA stability of the *fecABCDE* operons in the two *fec* system variants, thus resulting in different *fecA* mRNA levels and iron uptake abilities.

## Conclusion

In the present study, we identified a novel conjugative plasmid, pEC41, whose backbone was almost identical to the multiple ARGs-bearing plasmid pCTX-M3, which is highly conjugative and is potentially transmissible between clinical and environmental bacterial species. pEC41 harbored a *fec* iron uptake system that was absent in pCTX-M3. The *fec* system increases the virulence and competitive fitness of *E. coli* in systemic infections and UTIs, respectively. Due to its properties of efficient and broad spread of both antimicrobial resistance and virulence genes, pEC41 deserves further study on its distribution in environmental and clinical bacteria and on the impact of these plasmids on public health.

## Data Availability Statement

The sequences of pEC41 are deposited in the GenBank repository, accession number MW548582.

## Ethics Statement

All of the animal experimental procedures were reviewed and approved by The Institutional Animal Care and Use Committee (IACUC) of National Cheng Kung University, Tainan City, Taiwan (approval no. 108130). The studies involving human participants and bacteria collection were reviewed and approved by The Institutional Reviewer Board (IRB) of National Cheng Kung University Hospital, Tainan City, Taiwan (approval no. A-ER-107-403 and A-ER-104-313). The patients/participants provided their written informed consent to participate in this study.

## Author Contributions

W-CH, M-YW, and S-HW carried out the experiments in this study. MH, Y-LC, and C-HT contributed to the study conception, planning experiments, data analysis, and interpretation. M-HL, M-FL, and Y-LC contributed the materials and technical supports of the animal experiments. J-JW, S-LJ, and J-LW participated in conceptualization and interpretation of the data. M-CW, W-HL, and C-HT contributed materials and technical support. W-CH, M-YW, S-HW, Y-LC, and C-HT wrote the manuscript. All authors contributed to the article and approved the submitted version.

## Conflict of Interest

The authors declare that the research was conducted in the absence of any commercial or financial relationships that could be construed as a potential conflict of interest.

## Footnotes

1 https://cge.cbs.dtu.dk/services/PlasmidFinder/
