# Supplementary material for: The Ferric Citrate Uptake System Encoded in a Novel blaCTX–M–3- and blaTEM–1-Harboring Conjugative Plasmid Contributes to the Virulence of Escherichia coli
Source: Front Microbiol. 2021 May 26;12:667782. doi: 10.3389/fmicb.2021.667782 (PMC8187952; doi:10.3389/fmicb.2021.667782)
Supplement: Supplementary file 1 [file Data_Sheet_1.docx]

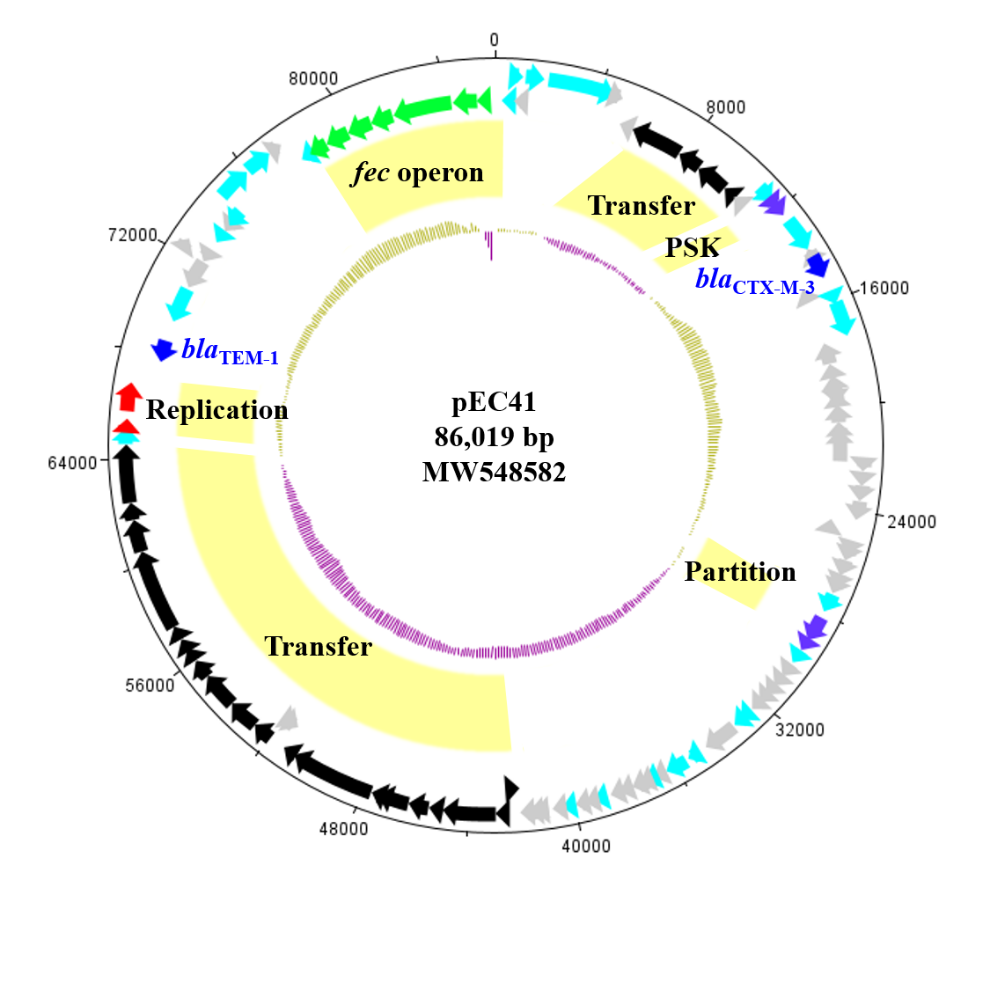
**Supplementary Figure S1**

**Supplementary Figure S1.** The map of pEC41. ORFs are denoted by arrows in the direction of transcription: ORFs homologous to genes with known functions are shown in light blue, and ORFs homologous to genes with unknown functions are shown in gray. The iron uptake system (*fec* system) is shown in green, whereas the antibiotic resistance genes (*bla*_CTX-M-3_ and *bla*_TEM-1_) are shown in blue. The conjugal transfer system (*tra* and *trb* genes) genes are presented in black. The partition (*parAB*) and postsegregational killing system (PSK; *pemIK*) genes are shown in purple, whereas the *repA* gene is shown in red. Functional sequence blocks are highlighted with shades of yellow, including the functions of conjugal transfer, plasmid stabilization (PSK and partition), and replication. The inner circle shows the GC plot (%). The map was generated using DNAPlotter software (Carver et al., 2009).

**References**

Carver, T., Thomson, N., Bleasby, A., Berriman, M., and Parkhill, J. (2009). DNAPlotter: circular and linear interactive genome visualization. *Bioinformatics* 25**,** 119-120.

**Supplementary Table S1. Primers used in this study.**

| Primers | Sequence (5’→3’) |
| --- | --- |
| Knockout of *fec* system in pEC41 | |
| UP-fecE-F | TCAGCAGAAGGATGTCGTCA |
| UP-fecE-R | CGAAGCAGCTCCAGCCTACACACCCATGTGTGTGGTGAAGTAGTCTGGCGAAGTCATAAG |
| DOWN-fecI-F | CGGAATAGGAACTAAGGAGGATATTCCTATCATGCGGAGTGCATCAAAAG |
| DOWN-fecI-R | CAGTCAGGTGGAACCCGAAATC |
|  |  |
| Knockout of *fec* system in MG1655 | |
| MG-UP-fec-F | CTGGCGCGACATCCGACGCC |
| MG-UP-fec-R | CCGTTTCCACGGTGTGCGTCCATGCGGAGTGCATCAAAAGTTAATTATCACGTAGTCATA |
| MG-DOWN-fec-F | GTAAATTGTCACAACGCCGCCCGATGTGCCTAATGAGGTAGATTGCACAGGCCGTAAGAA |
| MG-DOWN-fec-R | AGAAACTGGATTAGCAATTC |
|  |  |
| Chloramphenicol resistance cassette | |
| New P1 | TGTGTAGGCTGGAGCTGCTTCG |
| New P2 | ATAGGAATATCCTCCTTAGTTC |
|  |  |
| Amplification the gaps between contigs of plasmid pEC41 | |
| Contig_1-F | ACCTCAATACCTTTGATGGTGGCGTAAGCC |
| Contig_1-B | ACCTCAATACCTTTGATGGTGGCGTAAGCC |
| Contig_2-F | TTTGTCAGTCCAAGGTTGATACCATCGGCC |
| Contig_2-B | GGATAGTGATGGCATAAGAAGCAGTGCTCC |
| Contig_4-F | CTAAAGGGCGGGGAGGATGTCAATAATAGC |
| Contig_4-B | CTAAAACAGGTGTGCATTACTCCACCTGGC |
| Contig_2-F | TTTGTCAGTCCAAGGTTGATACCATCGGCC |
|  |  |
| Real-time PCR | |
| fecA-RT-2F | TCAATCAGTATGCCGCACAC |
| fecA-RT-2R | TTTCCCAGCGGTTTTACCTG |
| ftsZ-RT-F | CAATGGAACTTACCAATGAC |
| ftsZ-RT-R | TGTTTTACGCAGCGCTTGTG |
|  |  |
| Distribution of *fecA* | |
| fecA-F | GACCTGGCGATGAACTTTGG |
| fecA-R | CATCCATGTTGCCGAGCGAAAC |

**Supplementary Table S2. The genes encoded in pEC41**

| **Code** | **Gene** | **Coordinate** | **Direction** | **Protein** |
| --- | --- | --- | --- | --- |
| pEC41_001 | *tnpA* | 264..719 | <= | Transposase |
| pEC41_002 | *orf1* | 750..977 | <= | ORF1 |
| pEC41_003 | *int* | 891..1058 | => | Integrase |
| pEC41_004 | *tnp* | 1133..1849 | => | Transposase |
| pEC41_005 | *tnp* | 1974..4523 | => | Transposase |
| pEC41_006 | *orf2* | 4699..4848 | => | ORF2 |
| pEC41_007 |  | 5078..5587 | <= | Transcriptional regulator |
| pEC41_008 | *trbC* | 5635..7722 | <= | TrbC |
| pEC41_009 | *trbB* | 7735..8685 | <= | TrbB |
| pEC41_010 | *trbA* | 8696..10,003 | <= | TrbA |
| pEC41_011 | *trbN* | 10,003..10,398 | <= | TrbN |
| pEC41_012 | *orf3* | 10,503..10,886 | <= | ORF3 |
| pEC41_013 | *tir* | 10,966..11,619 | => | Tir |
| pEC41_014 | *pemI* | 11,712..11,969 | => | PemI |
| pEC41_015 | *pemK* | 11,971..12,303 | => | PemK |
| pEC41_016 | *tnp* | 12,598..13,860 | => | Transposase |
| pEC41_017 | *orf4* | 13,841..13,963 | => | ORF4 |
| pEC41_018 | *bla*_CTX-M-3_ | 14,195..15,070 | => | β lactamase CTX-M-3 |
| pEC41_019 | *orf5* | 15,117..15,665 | <= | ORF5 |
| pEC41_020 | *mucA* | 15,703..16,137 | => | MucA |
| pEC41_021 | *mucB* | 16,086..17,390 | => | MucB |
| pEC41_022 |  | 17,413..18,261 | <= | DNA polymerase III subunit epsilon |
| pEC41_023 | *orf6* | 18,264..18,584 | <= | ORF6 |
| pEC41_024 | *orf7* | 18,729..19,421 | <= | ORF7 |
| pEC41_025 | *orf8* | 19,436..19,645 | <= | ORF8 |
| pEC41_026 | *orf9* | 19,648..19,866 | <= | ORF9 |
| pEC41_027 | *orf10* | 19,911..20,594 | <= | ORF10 |
| pEC41_028 |  | 20,591..20,863 | <= | transcriptional regulator |
| pEC41_029 | *orf11* | 20,881..22,155 | <= | ORF11 |
| pEC41_030 | *orf12* | 22,346..22,495 | => | ORF12 |
| pEC41_031 | *orf13* | 22,682..23,038 | => | ORF13 |
| pEC41_032 | *orf14* | 23,016..23,600 | => | ORF14 |
| pEC41_033 | *orf15* | 23,597..24,316 | => | ORF15 |
| pEC41_034 | *orf16* | 24,478..25,023 | <= | ORF16 |
| pEC41_035 | *orf17* | 25,164..25,625 | => | ORF17 |
| pEC41_036 |  | 25,622..25,870 | => | transcriptional regulator |
| pEC41_037 | *orf18* | 25,863..26,450 | => | ORF18 |
| pEC41_038 | *orf19* | 26,447..26,932 | => | ORF19 |
| pEC41_039 | *orf20* | 26,929..27,177 | => | ORF20 |
| pEC41_040 | *resD* | 27,196..27,924 | => | ResD |
| pEC41_041 | *parA* | 28,165..29,139 | => | ParA |
| pEC41_042 | *parB* | 29,142..29,585 | => | ParB |
| pEC41_043 | *nucC* | 29,595..30,146 | => | endonuclease NucC |
| pEC41_044 | *orf21* | 30,264..30,770 | => | ORF21 |
| pEC41_045 | *orf22* | 30,763..31,242 | => | ORF22 |
| pEC41_046 | *orf23* | 31,271..31,681 | => | ORF23 |
| pEC41_047 | *orf24* | 31,799..32,062 | => | ORF24 |
| pEC41_048 | *orf25* | 32,084..32,446 | => | ORF25 |
| pEC41_049 | *radC* | 32,568..33,017 | => | DNA repair protein RadC |
| pEC41_050 |  | 33,062..33,328 | => | Transcriptional regulator |
| pEC41_051 |  | 33,392..34,675 | => | DGQHR domain-containing protein |
| pEC41_052 | *ccgA1* | 35,281..35,460 | => | CcgA1 |
| pEC41_053 |  | 35,533..36,393 | => | Methyltransferase |
| pEC41_054 | *orf26* | 36,583..36,921 | => | ORF26 |
| pEC41_055 | *rmoA* | 37,019..37,249 | => | RmoA |
| pEC41_056 | *orf27* | 37,362..37,475 | => | ORF27 |
| pEC41_057 | *orf28* | 37,547..37,783 | => | ORF28 |
| pEC41_058 | *orf29* | 37,868..38,278 | => | ORF29 |
| pEC41_059 | *orf30* | 38,340..38,645 | => | ORF30 |
| pEC41_060 | *klcA* | 38,846..39,286 | => | Antirestriction protein KlcA |
| pEC41_061 | *orf31* | 39,330..39,614 | => | ORF31 |
| pEC41_062 | *orf32* | 39,768..39,989 | => | ORF32 |
| pEC41_063 | *ssb* | 40,061..40,495 | => | Single-stranded DNA binding protein |
| pEC41_064 | *orf33* | 40,553..40,864 | => | ORF33 |
| pEC41_065 | *orf34* | 40,997..41,533 | => | ORF34 |
| pEC41_066 | *orf35* | 41,603..41,743 | => | ORF35 |
| pEC41_067 | *orf36* | 41,838..42,074 | => | ORF36 |
| pEC41_068 | *mobC* | 42,034..42,399 | <= | MobC |
| pEC41_069 | *mobB* | 42,675..42,992 | => | MobB/NikA |
| pEC41_070 | *mobA* | 42,979..44,958 | => | MobA/NikB |
| pEC41_071 | *traH* | 44,972..45,472 | => | TraH |
| pEC41_072 | *traI* | 45,469..46,248 | => | TraI |
| pEC41_073 | *traJ* | 46,259..47,422 | => | TraJ |
| pEC41_074 | *traK* | 47,412..47,672 | => | TraK |
| pEC41_075 |  | 47,697..50,864 | => | DNA primase |
| pEC41_076 | *traL* | 50,830..51,342 | => | TraL |
| pEC41_077 | *orf37* | 51,343..51,540 | <= | ORF37 |
| pEC41_078 | *orf37* | 51,527..51,937 | <= | ORF38 |
| pEC41_079 | *traM* | 51,936..52,718 | => | TraM |
| pEC41_080 | *traN* | 52,727..53,878 | => | TraN |
| pEC41_081 | *traO* | 53,890..55,239 | => | TraO |
| pEC41_082 | *traP* | 55,251..55,955 | => | TraP |
| pEC41_083 | *traQ* | 55,979..56,509 | => | TraQ |
| pEC41_084 | *traR* | 56,526..56,915 | => | TraR |
| pEC41_085 | *orf39* | 56,961..57,455 | => | ORF39 |
| pEC41_086 | *traU* | 57,452..60,502 | => | TraU |
| pEC41_087 | *traW* | 60,499..61,707 | => | TraW |
| pEC41_088 | *traX* | 61,704..62,354 | => | TraX-like protein |
| pEC41_089 | *traY* | 62,347..64,527 | => | TraY |
| pEC41_090 | *excA* | 64,530..65,183 | => | ExcA |
| pEC41_091 | *orf40* | 65,258..65,488 | => | ORF40 |
| pEC41_092 | *repA* | 65,774..66,841 | => | RepA |
| pEC41_093 | *bla*_TEM-1_ | 67,845..68,705 | <= | β lactamase TEM-1 |
| pEC41_094 | *tnp* | 69,526..70,896 | <= | Transposase |
| pEC41_095 | *orf41* | 71,011..72,147 | <= | ORF41 |
| pEC41_096 | *orf42* | 72,198..72,443 | <= | ORF42 |
| pEC41_097 | *orf43* | 72,449..72,640 | => | ORF43 |
| pEC41_098 |  | 73,122..73,664 | <= | Tunicamycin resistance protein |
| pEC41_099 | *Δaac(3)III* | 73,677..73,904 | <= | truncated aac(3)-III |
| pEC41_100 | *tnpA* | 73,957..74,673 | <= | TnpA IS26 |
| pEC41_101 | *tnp* | 74,652..75,917 | => | Transposase |
| pEC41_102 | *tnp* | 75,996..77,000 | => | Transposase |
| pEC41_103 | *orf44* | 77,019..77,141 | <= | ORF44 |
| pEC41_104 |  | 77,355..77,540 | => | cyclic diguanylate phosphodiesterase (EAL) domain |
| pEC41_105 | *tnp* | 77,871..78,278 | <= | Transposase |
| pEC41_106 | *fecE* | 78,263..79,030 | <= | FecE |
| pEC41_107 | *fecD* | 79,031..79,987 | <= | FecD |
| pEC41_108 | *fecC* | 79,984..80,982 | <= | FecC |
| pEC41_109 | *fecB* | 80,979..81,881 | <= | FecB |
| pEC41_110 | *fecA* | 81,926..84,250 | <= | FecA |
| pEC41_111 | *fecR* | 84,336..85,289 | <= | FecR |
| pEC41_112 | *fecI* | 85,286..85,807 | <= | FecI |
